# Impaired acid stress resistance in *Salmonella* Typhi Ty2

**DOI:** 10.1128/jb.00197-26

**Published:** 2026-09-23

**Authors:** Kiranmai Joshi, Wai Yee Fong, Marie-Pierre Blanc, Fermin E. Guerra, Ferric C. Fang

**Affiliations:** 1 Department of Laboratory Medicine and Pathology, University of Washington 7284, Seattle, Washington, USA; 2 Department of Microbiology, University of Washington 7284, Seattle, Washington, USA; University of Virginia School of Medicine, Charlottesville, Virginia, USA

**Keywords:** membrane proteins, pseudogenes, stress response, RNA-seq, acid resistance

## Abstract

*Salmonella enterica* encounters acid stress during gastrointestinal transit and within the phagosomal environment of macrophages. Acid stress resistance has been well characterized in *Salmonella enterica* serovar Typhimurium, but comparative studies in the human-adapted *Salmonella enterica* serovar Typhi are limited. We compared the growth of *S*. Typhimurium 14028s and *S*. Typhi Ty2 at pH values ranging from 3 to 8 and observed that *Salmonella enterica* serovar Typhimurium exhibits enhanced growth at pH 4.5 compared to *S*. Typhi. Comparative transcriptomic profiling of *S*. Typhimurium and *S*. Typhi at pH 4.5 and 7.5 identified numerous differentially expressed acid-induced genes (DEGs), including genes encoding membrane proteins (OmpC, PhoE, HydB), a transcriptional regulator (RpoS), and stress response proteins (YciG, STM14_1829, YmdF). Targeted deletion of selected genes in *S*. Typhimurium significantly suppressed growth at acidic pH, confirming their role in acid stress resistance. These resistance mechanisms are compromised in *S*. Typhi due to pseudogenization. Heterologous expression of pseudogenized genes in *S*. Typhi restored acid tolerance. Collectively, these findings suggest that *S*. Typhi has lost the ability to withstand acid stress due to genomic decay and the loss of multiple genes essential for acid survival in *S*. Typhimurium, reflecting divergent evolutionary paths in these two serovars.

**IMPORTANCE:**

*Salmonella* Typhimurium must adapt to acidic pH conditions in the intestinal tract and the intracellular environment to cause infection. In this study, we show that the enteric fever serovar *Salmonella* Typhi exhibits impaired growth at pH 4.5 in comparison to *Salmonella* Typhimurium. We further show that the loss of specific membrane proteins, a transcriptional regulator, and a family of stress response proteins in *Salmonella* Typhi are responsible for this difference. Collectively, these observations suggest that *Salmonella* Typhi has evolutionarily lost the ability to withstand acid stress due to differences in its interaction with the human host. This has important implications for the pathogenesis of typhoid fever.

## INTRODUCTION

*Salmonella enterica* poses a significant global health burden as a leading cause of foodborne illness, resulting in ~93 million cases of gastroenteritis and ~14 million cases of enteric fever annually (1, 2). Although most non-typhoidal *Salmonella* infections are self-limiting, severe infections can lead to hospitalization and death, particularly in immunocompromised individuals or those at the extremes of age (3). The ubiquity of this pathogen, its ability to be transmitted between vertebrate hosts and the environment, and the increasing prevalence of antimicrobial-resistant strains have made it a global health priority (4). The disease burden of salmonellosis is highest in low- and middle-income countries largely due to poor sanitation, but frequent foodborne outbreaks also occur in high-income countries (5).

As a foodborne pathogen, *Salmonella* encounters multiple stresses in the environment and within an infected host, including osmotic shock, high temperature, oxidative stress, nitrosative stress, extreme pH, and nutrient deprivation (6). To survive these conditions, *Salmonella* has evolved specific adaptive responses. Resistance to low pH is an adaptive mechanism of enteric pathogens that is crucial for their survival (7, 8). The acid resistance systems AR1, AR2, and AR3 of *Escherichia coli* have been extensively characterized (9). Amino acid decarboxylases play a crucial role in acid resistance of *E. coli* and *Salmonella* by consuming intracellular protons to regulate cytosolic pH (10, 11). Although *Salmonella* is less acid resistant than *E. coli*, it exhibits an acid tolerance response (ATR) involving the regulated synthesis of acid shock proteins controlled by multiple regulators, including RpoS (σ^S^, σ^38^), Fur, and the PhoPQ two-component system (12–14). The *Salmonella* ATR can be triggered by modest reductions in pH, which subsequently protects bacteria from more severe degrees of acid stress (15). Although earlier observations suggest that the non-typhoidal serovar *S*. Typhimurium and the enteric fever serovar *S*. Typhi may differ with respect to the ATR and extreme acid stress tolerance (pH 3.3) (16), the mechanistic basis for these differences has not been investigated.

*Salmonella enterica* serovars are categorized as typhoidal or non-typhoidal, a distinction based on host range and clinical characteristics. Most non-typhoidal *Salmonella* serovars (e.g., *S*. Typhimurium and *S*. Enteritidis) infect a broad range of vertebrate hosts and cause localized gastrointestinal disease in humans and systemic infections in animals, whereas enteric fever serovars (e.g., *S*. Typhi and *S*. Paratyphi) are restricted to human hosts, in which they cause systemic infections (17). In contrast to the self-limiting nature of non-typhoidal *Salmonella* gastroenteritis, typhoid fever necessitates antimicrobial therapy, which has been complicated by the emergence of drug-resistant strains (2, 18). Despite sharing over 90% genomic identity, non-typhoidal and typhoidal serovars of *Salmonella* are distinguished by numerous genetic differences, including pseudogenes, unique genes, and horizontally acquired genes (19, 20). Both non-typhoidal and enteric fever serovars are anticipated to encounter acid stress during the host-pathogen interaction: within the stomach, in the distal intestine, and within the phagolysosomes of professional phagocytic cells.

In the present study, the growth of non-typhoidal *S*. Typhimurium 14028s and typhoidal *S*. Typhi Ty2 was compared across a range of pH conditions (pH 3–8). RNA-Seq was performed to analyze the differential gene expression in *S*. Typhimurium 14028s and *S*. Typhi Ty2 at neutral (7.5) or acidic (4.5) pH conditions. Differentially expressed genes (DEGs) and pseudogenes were analyzed to determine their contribution to the acid resistance phenotypes of *S*. Typhimurium and *S*. Typhi.

## RESULTS

### *S*. Typhi exhibits impaired growth at pH 4.5 compared to *S*. Typhimurium

**Fig 1:**
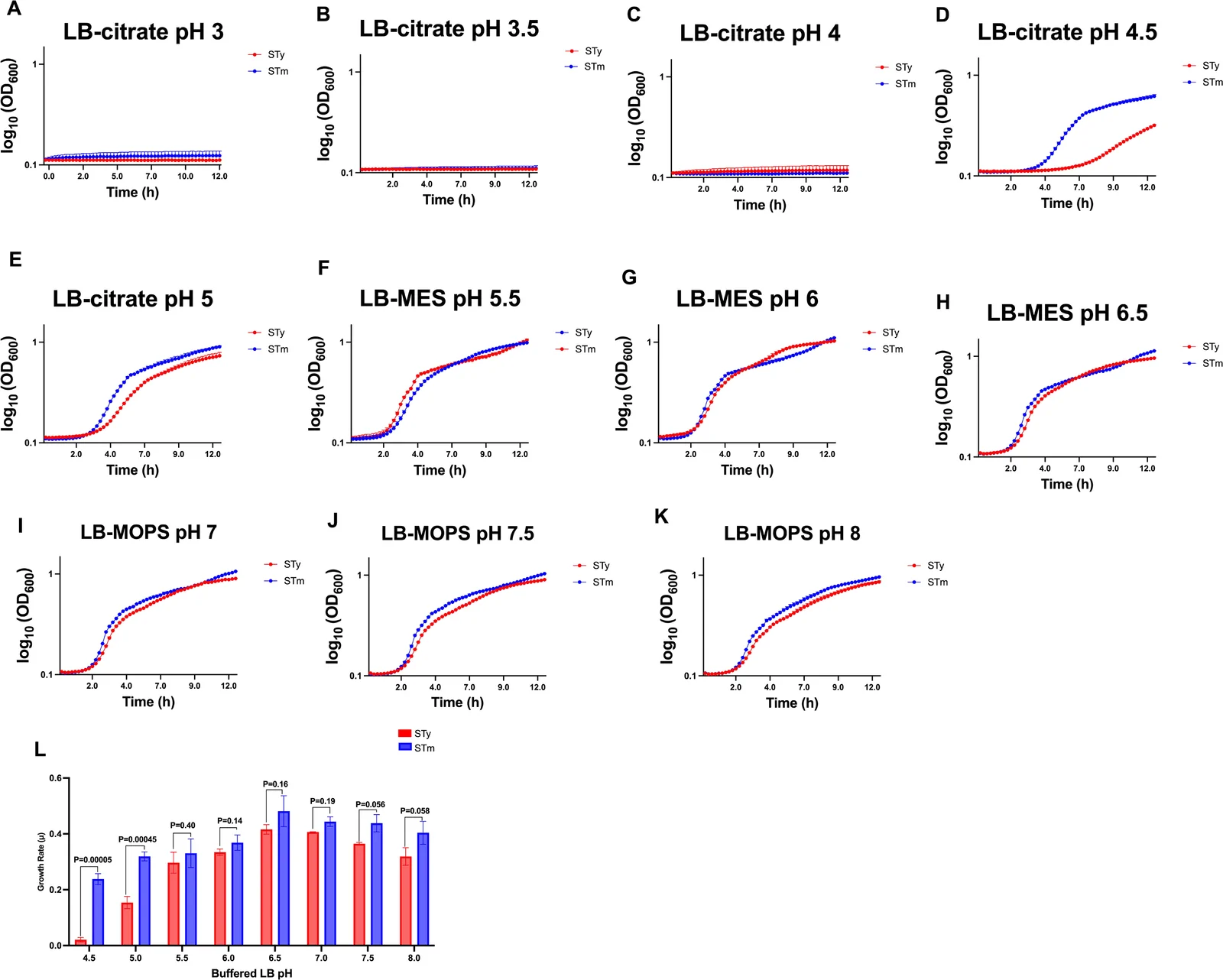
Growth of *S*. Typhimurium and *S*. Typhi in buffered LB media. Optical density was monitored for S. Typhimurium 14028s (STm-Blue) and S. Typhi Ty2 (STy-Red) grown at various pH conditions. Overnight cultures of STm and STy were diluted to 0.001 OD_600_ in LB buffered with citrate (for pH 3.0, 3.5, 4.0, 4.5, 5.0) (**A–E**), MES (for pH 5.5, 6.0, 6.5) (**F–H**), or MOPS (for pH 7.0, 7.5, 8.0) (**I–K**), and OD_600_ was measured every 15 min at 37°C for 12 h in a Bioscreen C. (**L**) Bar graphs represent the mean slopes of exponential growth of STm and STy in buffered LB at pH 4.5–8.0, with error bars representing the standard deviation from three independent assays. Statistical analysis was performed in GraphPad Prism 10.0. *P* values were determined using an unpaired two-tailed *t*-test.

Growth of *S*. Typhimurium or *S*. Typhi was monitored by measuring the optical density at 600 nm every 15 min in LB broth buffered at pH values ranging from 3 to 8 at 0.5 intervals over a 12 h period on a Bioscreen C (Growth Curves, Ltd., Finland). No growth of either serovar was observed at pH 3.0, 3.5, or 4.0 (Fig. 1A through C). Growth at pH 4.5 was significantly attenuated for *S*. Typhi compared to *S*. Typhimurium, whereas no significant difference in the growth of the two serovars was observed at pH 5–8, except for mild suppression of *S*. Typhi at pH 5.0 (Fig. 1D through L).

### Numerous genes are differentially expressed in *S*. Typhimurium and *S*. Typhi at pH 4.5

**Fig 2:**
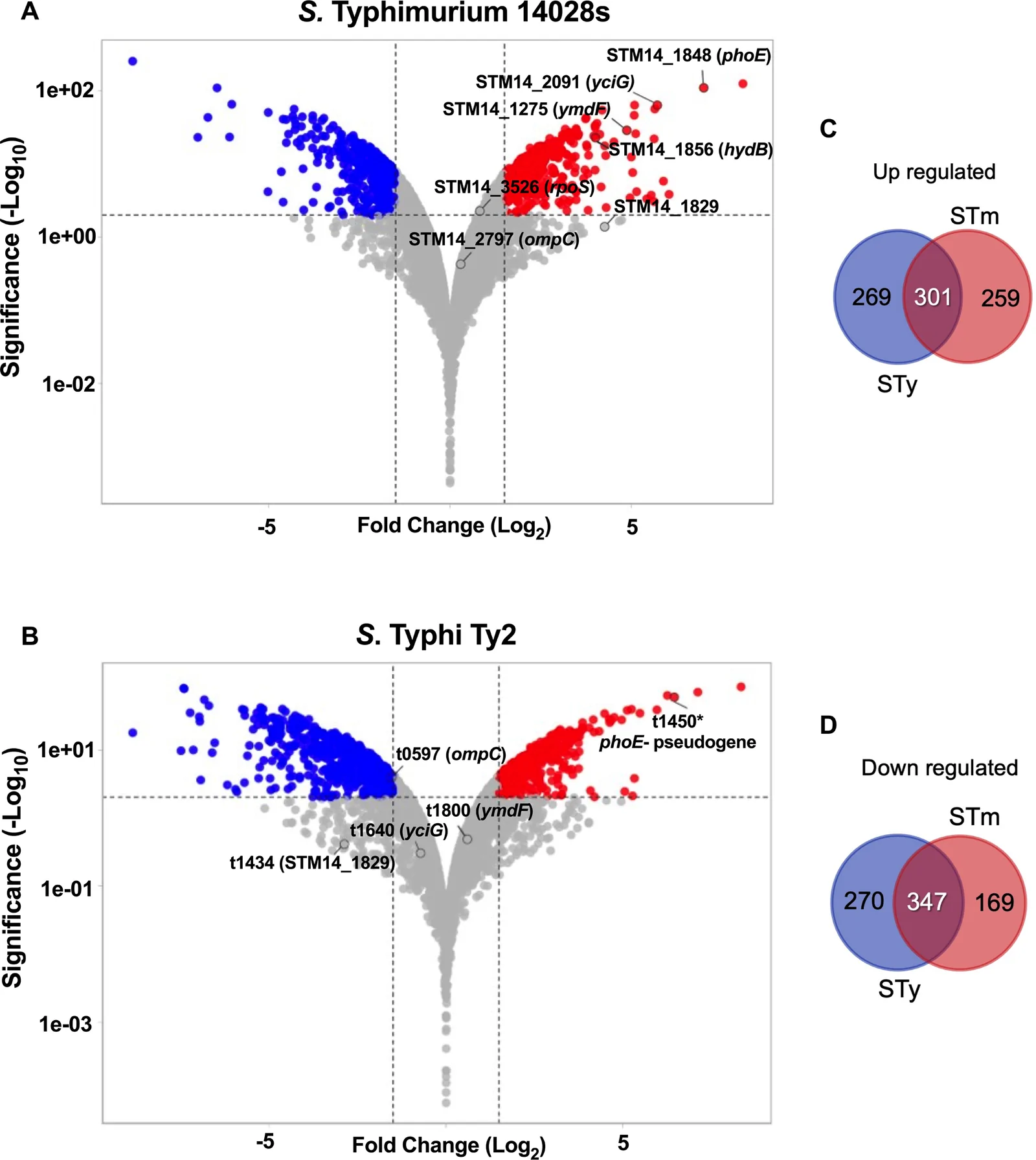
Transcriptomic analysis of *S*. Typhimurium and *S*. Typhi. Gene expression was analyzed by RNA-Seq in *S*. Typhimurium 14028s (STm) and *S*. Typhi Ty2 (STy) grown to early log phase (OD_600_ 0.3) in LB medium at pH 4.5 or 7.5. Differential gene expression in both strains was analyzed using the FeatureCounts read-counting program. Volcano plots show differential gene expression in STm (**A**) and STy (**B**), highlighting the target genes in the current study. Genes significantly upregulated or downregulated at pH 4.5 relative to pH 7.5 (log2FC > 1, adj-*P* ≤ 0.05) are indicated in red or blue, respectively. (**C and D**) Venn diagrams represent the number of upregulated (**C**) and downregulated (**D**) genes in STm (red) and STy (blue), respectively.

To identify the genetic differences responsible for greater resistance of *S*. Typhimurium to acid stress at pH 4.5, the *Salmonella* gene expression was compared at pH 4.5 and 7.5 by RNA-Seq. Total RNA was isolated from *S*. Typhimurium 14028s or *S*. Typhi Ty2 at early log phase (OD_600_ 0.3) under acidic (4.5) and neutral (7.5) pH conditions, and RNA-Seq was performed. Transcripts were quantified using the FeatureCounts read-counting program. Annotation of transcriptional profiles against the GenBank database yielded 4,849 genes from *S*. Typhimurium and 4,704 genes from *S*. Typhi. Comparison of gene expression in *S*. Typhimurium 14028s and *S*. Typhi Ty2 at acidic and neutral pH revealed multiple differentially expressed genes at pH 4.5 compared to pH 7.5, including both distinct and common sets for each serovar. A total of 645 genes and 632 genes were upregulated, and a total of 665 and 831 genes were downregulated in *S*. Typhimurium and *S*. Typhi, respectively, at pH 4.5 relative to expression at pH 7.5 (log2FC > 1, adj-*P* ≤0.05). However, considerable differences were observed between the serovars, with 259 *S*. Typhimurium genes upregulated at pH 4.5 relative to *S*. Typhi. The volcano plots and the Venn diagrams in Fig. 2A through D represent the number of differentially expressed genes in *S*. Typhimurium and *S*. Typhi at acidic pH 4.5, and Tables S1 and S2 show the list of all the DEGs in *S*. Typhimurium, highlighting the pseudogenes (green rows) of *S*. Typhi (21).

Some of the genes that were differentially expressed encoded membrane proteins and stress response proteins. Differentially expressed outer membrane protein (OMP) genes included *ompC*, *ompF*, *phoE*, and *ompD*. Under acid pH conditions, *S*. Typhimurium exhibited upregulation of the general stress response genes *yciG*, *STM14_1829*, and *ymdF*, which encode small intrinsically disordered proteins (22), whereas these genes were downregulated in *S*. Typhi (Table S3). Expression of genes encoding transcriptional regulators involved in acid stress responses, such as *phoPQ*, *fur*, *envZ*, and *ompR*, revealed no difference in expression between the two serovars, with the exception of *rpoS*, which was slightly upregulated in *S*. Typhimurium but is non-functional in the *S*. Typhi Ty2 strain due to a frameshift mutation (23).

Specific attention was paid to genes encoding enzymes with known roles in acid resistance, including *narWV*, *fdhN*, *hydB*, *adiA*, *adiC*, *speC*, *speF*, and *cadA* (24, 25). A significant upregulation of *narWV*, *fdhN*, *hydB*, and *speC* was observed in *S*. Typhimurium, whereas these genes are pseudogenes in *S*. Typhi (26). The *adiA*, *adiC*, *speF*, and *cadA* genes did not show differential expression at pH 4.5 in *S*. Typhimurium and *S*. Typhi (Table S3).

### *S*. Typhi lacks multiple membrane proteins that contribute to *S*. Typhimurium acid stress resistance

**Fig 3:**
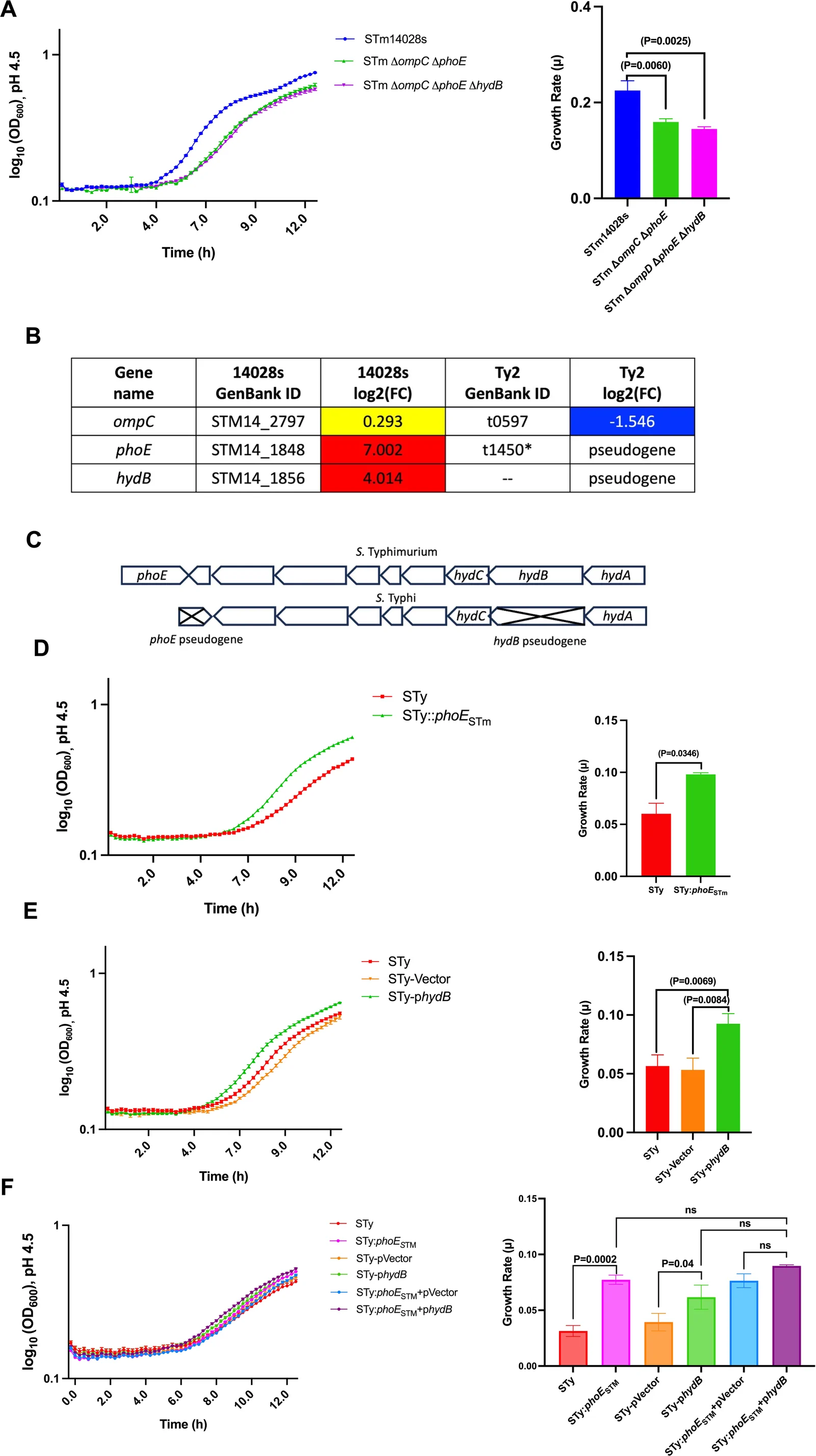
*S.* Typhi lacks multiple membrane proteins that contribute to *S.* Typhimurium acid stress resistance. The porin PhoE and membrane-bound hydrogenase HydB are present in *S*. Typhimurium (STm) but not in *S*. Typhi (STy). (**A**) Growth of wt STm, STm *ΔompC ΔphoE,* and STm *ΔompC ΔphoE ΔhydB* in buffered LB at pH 4.5. (**B**) Fold change of OMPs (*ompC, phoE*) and hydrogenase (*hydB*) gene expression in LB buffer at pH 4.5 in STm and STy. (**C**) Gene maps of STm and STy derived from BioCyc database showing the pseudogenes *phoE* and *hydB*. (**D**) *phoE* of STm was allelic-exchanged in STy and growth monitored for STm wt and STy*Δt1450::phoE_STm_* in buffered LB at pH 4.5. (**E**) Growth of STm wt, STy-Vector (pJK770), and STy-p*hydB* (pJK770-hydB) in buffered LB at pH 4.5. (**F**) Growth of STy wt, STy::*phoE_STm_*, STy-Vector (pJK770), STy-p*hydB* (pJK770-hydB), STy::*phoE_STm_* + Vector (pJK770), and STy::*phoE_STm_* + p*hydB* (pJK770-hydB) in buffered LB at pH 4.5. To generate the growth curves, bacteria were cultured in LB-citrate (pH 4.5) or LB-MOPS (pH 7.5), and OD_600_ measured every 15 min at 37°C for 12 h on a Bioscreen C. Typical representative growth curve of three independent experiments is shown. Statistical analysis was performed in GraphPad Prism 10.0. Bar graphs represent the mean slopes of exponential growth, with error bars representing the standard deviation from three independent assays. *P* values were determined using an unpaired two-tailed *t*-test (**A, D, and E**). ns, non-significant.

Individual deletion mutations were constructed in differentially expressed genes encoding outer membrane proteins (OMPs) in *S*. Typhimurium. Growth of mutant strains was compared to that of wild-type *S*. Typhimurium and *S*. Typhi at pH 4.5 and 7.5. Mutants containing single OMP gene deletions (STm Δ*ompC*, *ΔompD*, *ΔompF*, or *ΔphoE*) exhibited growth phenotypes similar to that of wild-type *S*. Typhimurium (Fig. S1A). Double gene deletions (STm Δ*ompC*Δ*ompF*, Δ*ompC*Δ*ompD*, Δ*ompD*Δ*phoE*, or Δ*ompF*Δ*phoE*) did not impair the growth of *S*. Typhimurium at pH 4.5, with the exception of the Δ*ompC*Δ*phoE* mutant, which had a detectable growth defect. Mutants containing triple gene deletions (STm Δ*ompC*Δ*ompD*Δ*phoE* and STm Δ*ompC*Δ*ompF*Δ*phoE*) also exhibited slower growth at pH 4.5 compared to the wild-type *S*. Typhimurium (Fig. 3A; Fig. S1B and C). No growth difference was observed between STm Δ*ompC*Δ*phoE* and wild-type S. Typhimurium at pH 7.5 (Fig. S1D). Collectively, these observations indicate that OmpC and PhoE make redundant contributions to acid stress resistance in *S*. Typhimurium. A notable correlation was observed between the growth kinetics and expression of *ompC* and *phoE* in the RNA-Seq data. Expression of *ompC* at pH 4.5 was higher in *S*. Typhimurium than in *S*. Typhi, and *phoE* was not expressed in *S*. Typhi because of truncation of its coding region (Fig. 3B and C). We performed an allelic exchange to replace the non-functional *phoE* gene (*t1450*) of *S*. Typhi with a functional *phoE* gene from *S*. Typhimurium. Growth curves were then analyzed under neutral and acidic pH conditions. The repaired STy Δ*t1450::phoE*_STm_ allele partially restored *S*. Typhi growth at pH 4.5, confirming the contribution of PhoE to *Salmonella* acid resistance (Fig. 3D). There was no difference in the growth phenotype of wild-type *S*. Typhi and the STy *t1450::phoE*_STm_ mutant at pH 7.5 (Fig. S1E).

HydB is a subunit of a membrane-bound Hyd enzyme complex that enables *S.* Typhimurium to use molecular hydrogen as an energy source during aerobic respiration (27). Upregulation of *hydB* was observed at pH 4.5 in *S.* Typhimurium, but *hydB* is predicted to be non-functional in *S.* Typhi due to a nonsense single-nucleotide polymorphism (SNP) (Fig. 3B and C). Although deletion of *hydB* alone had a minimal effect on growth of *S.* Typhimurium at pH 4.5, an additive effect on growth was observed when a *ΔhydB* mutation was introduced into a *S.* Typhimurium Δ*ompC*Δ*phoE* double-mutant strain (Fig. 3A; Fig. S1F). Growth of the triple Δ*ompC*Δ*ophoE*Δ*hydB* mutant was unaffected at pH 7.5 (Fig. S1D). Furthermore, heterologous constitutive expression of the *hydB* gene of *S.* Typhimurium in *S.* Typhi on an expression vector enhanced the growth of *S.* Typhi at pH 4.5 compared to vector alone or wild-type *S*. Typhi (Fig. 3E). No difference in growth of *S.* Typhi carrying p*hydB*, *S.* Typhi with the vector alone, or wild-type *S.* Typhi was observed at pH 7.5 (Fig. S1G). Furthermore, a double-complemented *S*. Typhi strain expressing both *phoE* and *hydB* was generated by heterologous constitutive expression of the *hydB* gene of *S.* Typhimurium in STy *t1450::phoE*_STm_ on an expression vector. STy *t1450::phoE*_STm_ + p*hydB* exhibited only a very modest, non-significant increase in growth rescue compared to the single-gene complementation strains STy *t1450::phoE*_STm_ and STy-p*hydB* at pH 4.5 and no difference in growth at pH 7.5 (Fig. 3F; Fig. S1H). Taken together, these results indicate that the outer membrane proteins OmpC and PhoE and the aerobic membrane-bound Hyd hydrogenase contribute to acid stress resistance in *S*. Typhimurium but are absent or deficient in *S*. Typhi

### *S.* Typhi lacks stress response genes that contribute to *S.* Typhimurium acid stress resistance

**Fig 4:**
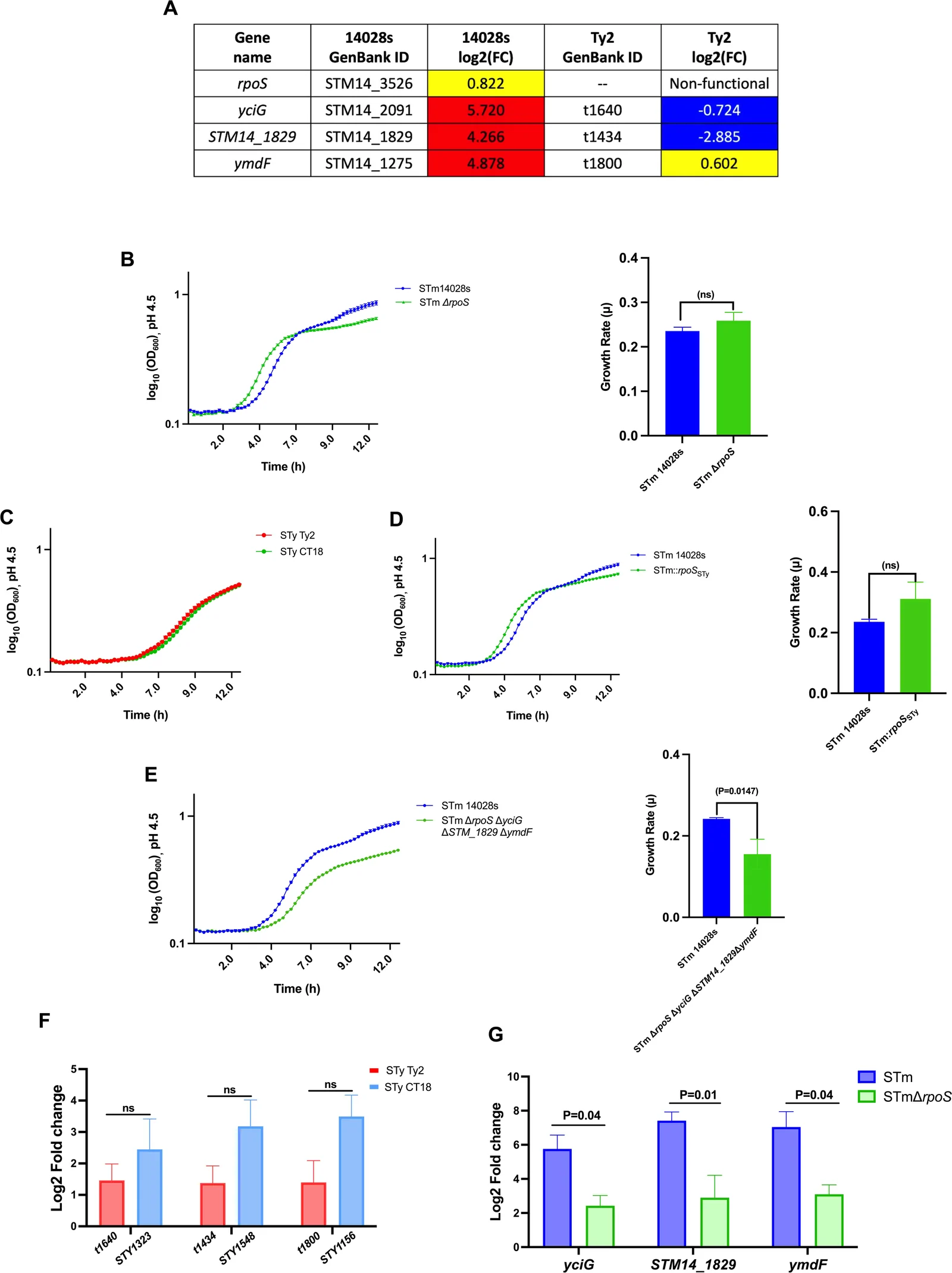
*S.* Typhi lacks stress response proteins that contribute to acid resistance in *S.* Typhimurium. Activity of the alternative sigma factor RpoS (σ^S^) and the small, intrinsically disordered proteins YciG, YmdF, and STM14_1829 is compromised in S. Typhi. (**A**) Fold change of *rpoS* and *yciG, STM14_1829*, and *ymdF* gene expression at pH 4.5 in STm and STy. S. Typhimurium strain that lacks *rpoS, yciG, STM14_1829*, and *ymdF* are susceptible to growth inhibition at acidic pH 4.5. (**B**) Growth of STm wt and STm *ΔrpoS* in buffered LB at pH 4.5. *rpoS* of STy was expressed in STm through allelic exchange, and growth was monitored. (**C**) Growth of STy Ty2 and STy CT18 in buffered LB at pH 4.5. (**D**) Growth of STm wt and STm::*rpoS_STy_* in buffered LB at pH 4.5. (**E**) Growth of STm wt and STm *ΔrpoS ΔyciG ΔSTM14_1829 ΔymdF* in buffered LB at pH 4.5. (**F**) Expression of IDPs *t1640, t1434*, and *t1800* in STy Ty2 and STY1323, STY1548, and STY1156 in STy CT18 (homologs of *yciG, STM14_1829*, and *ymdF* in STm, respectively) grown to early log phase in buffered LB at pH 4.5 and 7.5 (**G**) Expression of *yciG, STM14_1829*, and *ymdF* in wt and *ΔrpoS* STm in buffered LB at pH 4.5 and 7.5. Transcript levels were quantified by qRT-PCR and normalized to housekeeping gene *gmk* and to transcript levels at pH 7.5. Data represent mean ± SD (*n* ≥ 3). To generate the growth curves, bacteria were grown in LB-citrate (pH—4.5) or LB-MOPS (pH—7.5) and OD_600_ measured every 15 min at 37°C for 12 h on a Bioscreen C. Typical growth curves representative of three independent experiments are shown. Statistical analysis was performed in GraphPad Prism 10.0. Bar graphs represent the mean slopes of exponential growth, with error bars representing the standard deviation from three independent assays. *P* values were determined using an unpaired two-tailed *t*-test (**B, C, and D**).

A complex network of transcriptional regulators is activated by external stresses, such as osmotic shock, oxidative stress, nitrosative stress, nutrient deprivation, and acid stress conditions, which allows *Salmonella* to withstand varying environmental conditions during infection (7). RNA-Seq analysis of *S.* Typhimurium and *S.* Typhi did not show differences in most of the key transcriptional regulators implicated in acid stress resistance, such as *phoPQ*, *fur*, *ompR*, or *envZ* (Table S3). However, the *rpoS* gene encoding the alternative sigma factor σ^38^ (RpoS, σ^S^) is non-functional in the *S.* Typhi Ty2 strain due to a frameshift mutation (14, 23) (Fig. 4A). We found that deletion of *rpoS* in *S.* Typhimurium resulted in a severe growth defect at pH 4.5 but not at pH 7.5, consistent with an important role in acid resistance (Fig. 4B; Fig. S2A). As *rpoS* is non-functional in *S.* Typhi Ty2, we investigated the ability of *S*. Typhi CT18, which contains an intact *rpoS* gene, at pH 4.5 and 7.5. Growth of *S*. Typhi CT18 remained defective at pH 4.5, suggesting that additional genes deficient in *S.* Typhi are required for acid stress resistance (Fig. 4C; Fig. S2B). In addition, the native *rpoS* gene of *S.* Typhimurium was replaced with the *rpoS* gene from *S.* Typhi Ty2 via allelic exchange. An altered growth phenotype was observed in the allelic-exchanged STm::*rpoS*_STy_ strain at acidic pH, with more rapid entry into exponential growth and reduced terminal OD_600_, confirming the impaired activity of σ^38^ in *S.* Typhi Ty2. Growth of the STm::*rpoS*_STy_ strain at pH 7.5 was unaltered compared to that of STm wt (Fig. 4D; Fig. S2C).

The alternative sigma factor σ^38^ (*rpoS*) serves as a master regulator coordinating the general stress response network in *Salmonella*, modulating the expression of genes crucial for maintaining cellular viability under a range of environmental stresses, including oxidative stress, nutrient deprivation, osmotic shock, and acid pH (28–30). The small, intrinsically disordered proteins YciG, YmdF, and STM14_1829 have been suggested to play a role in the general stress response and exhibit redundant effects on flagellar synthesis in *S.* Typhimurium (22). Reduced expression of these genes at pH 4.5 in *S.* Typhi is most likely attributable to regulation by σ^38^ (Fig. 4A) (31). *S.* Typhimurium mutants carrying single deletions of *yciG*, *STM14_1829*, or *ymdF* exhibited growth phenotypes similar to that of wild-type *S*. Typhimurium at pH 4.5 (Fig. S2D). Modestly impaired growth of mutant *S.* Typhimurium carrying triple (Δ*yciG*Δ*STM14_1829*Δ*ymdF*) or double gene deletions (Δ*yciG*Δ*ymdF*) was observed, but other double gene deletions (Δ*yciG*Δ*STM14_1829* and Δ*STM14_1829*Δ*ymdF*) did not affect growth at acid pH relative to wild-type *S.* Typhimurium (Fig. S2E). A quadruple *S.* Typhimurium deletion mutant lacking all three intrinsically disordered proteins as well as *rpoS* (Δ*rpoS*Δ*yciG*Δ*STM14_1829*Δ*ymdF*) exhibited reduced growth at pH 4.5 with moderate growth suppression at pH 7.5 compared to wild-type STm (Fig. 4E; Fig. S2F), suggesting that additional σ^38^-dependent loci contribute to acid resistance.

Despite possessing a functional *rpoS* allele, *S*. Typhi CT18 exhibited an acid-sensitive growth phenotype similar to that of the *rpoS*-deficient *S*. Typhi Ty2 strain. To investigate further, transcriptional profiles of the RpoS-regulated small intrinsically disordered general stress response genes *t1640*, *t1434*, and *t1800* in *S.* Typhi Ty2 and *STY1323*, *STY1548*, and *STY1156* in *S.* Typhi CT18, which are homologs of *yciG*, *STM14_1829*, and *ymdF* in *S.* Typhimurium, were obtained by quantitative real-time PCR (qRT-PCR) under pH 4.5 conditions. As predicted by earlier transcriptomic data, these IDPs were poorly expressed in *S*. Typhi Ty2 at pH 4.5. Although *S*. Typhi CT18 carries a wild-type *rpoS* gene, expression of these target genes was only slightly higher than in Ty2, suggesting that there may be additional deficiencies in the network of factors controlling the RpoS activity in *S.* Typhi (Fig. 4F). Conversely, a Δ*rpoS* mutation completely abolished the induction of *yciG*, *STM14_1829*, and *ymdF* in *S.* Typhimurium at pH 4.5 (Fig. 4G). This confirms the presence of an intact RpoS signaling cascade in *S*. Typhimurium, which underpins its robust acid resistance.

## DISCUSSION

The present study demonstrates reduced survival of *S.* Typhi at acidic pH (pH 4.5) compared to *S.* Typhimurium. This phenotype is biologically relevant, as *Salmonella* is reported to encounter comparable levels of acid stress in the intestinal tract and within the phagolysosomes of phagocytic cells (32–34). We compared the transcriptional response *of S*. Typhi *and S*. Typhimurium at pH 4.5 and identified multiple genetic determinants required for acid stress resistance in *S.* Typhimurium that are deficient in *S.* Typhi.

The acid tolerance response has been extensively studied in *S.* Typhimurium but not in *S.* Typhi (15). Mendoza-Mejía et al. reported that the deletion of transcriptional regulators (MarR, MarA, MarB), Hya hydrogenase components, or fimbrial proteins increases the acid sensitivity of *S.* Typhi (35). However, they did not investigate the basis for the relative acid sensitivity of *S.* Typhi in comparison to *S.* Typhimurium. In the present study, we measured the genome-wide transcriptional response of *S.* Typhimurium and *S.* Typhi grown in LB broth at neutral (pH 7.5) or acidic (pH 4.5) conditions using RNA-Seq and identified significant differences in the transcriptional response of these two serovars at pH 4.5. While *S.* Typhimurium exhibits the induction of numerous acid-responsive genes, many orthologous genes are either suppressed or pseudogenized in *S.* Typhi.

An extensive literature describes the importance of amino acid decarboxylases in the acid resistance of *Salmonella*, which convert amino acids into corresponding amines to maintain cytosolic pH. The arginine and lysine decarboxylation systems encoded by *cadA* and *adiC*, respectively, have been implicated in the acid response of *Salmonella* (24, 25). Consistent with previous findings, we observed *cadA* and *adiC* induction in both *S.* Typhimurium and *S.* Typhi under acidic conditions. Deletion of these genes decreased growth at acidic pH (data not shown), suggesting a conserved acid resistance mechanism in both serovars. In *S.* Typhimurium, despite the pH-dependent induction of *speF* and *speC*, which encode the ornithine decarboxylase system, deletion of these genes did not affect survival at pH 4.5 (data not shown). In *S.* Typhi, the ornithine decarboxylase system is non-functional due to pseudogenization of the *speC* gene and a lack of induction of the *speF* expression (Table S3).

We investigated the role of membrane proteins in the cellular stress response, as they maintain homeostasis by acting as sensors for downstream signaling cascades and transporters of substrates across the bacterial membrane (36). Outer membrane proteins (OMPs) contribute to stress responses in enteric bacteria, including *E. coli*, *Yersinia*, and *Shigella*, and may exhibit up or downregulation under environmental stress (37). OmpC and OmpF have been reported to be required for acid stress resistance in *E. coli* (38). However, PhoE was the most strongly induced OMP in *S.* Typhimurium at pH 4.5 and a pseudogene in *S.* Typhi (Fig. 3C). Deletion mutants of *ompC*, *ompD*, *ompF*, and *phoE* were created in *S.* Typhimurium, and growth curve assays demonstrated the essentiality of *ompC* and *phoE* for *S.* Typhimurium growth at pH 4.5 (Fig. 3A). Deficient *ompC* expression and pseudogenization of *phoE* in *S.* Typhi appear to contribute to the deficient acid resistance of *S.* Typhi. In addition, the expression of the membrane-bound periplasmic-facing hydrogenase (*hyd*) operon was observed in *S.* Typhimurium at pH 4.5, but *hydB* is a pseudogene in *S.* Typhi. This aerobic hydrogenase converts protons to molecular hydrogen, which raises cytoplasmic pH (39). Introducing the cloned *S.* Typhimurium *phoE* or *hydB* genes into *S.* Typhi partially restored growth at pH 4.5, confirming the contribution of these pseudogenes to the acid sensitivity of *S.* Typhi (Fig. 3D and E). Complementation of both *phoE* and *hydB* in *S*. Typhi yielded only a very slight additive restoration of growth at pH 4.5, which did not achieve statistical significance (Fig. 3F). This incomplete phenotypic restoration implies that additional genetic differences contribute to the different levels of acid stress resistance in *S*. Typhimurium and *S.* Typhi.

Comparison of transcriptional regulator expression in *S.* Typhimurium and *S.* Typhi at acid and neutral pH did not reveal differential expression of known key regulators of acid stress resistance, including *phoPQ*, *fur*, *envZ*, and *ompR* (12, 13, 40). However, *rpoS* (σ^S^, σ^38^) is known to be non-functional in *S.* Typhi Ty2, as well as in approximately one-third of natural *S.* Typhi isolates (41), and has been shown to regulate the acid tolerance response in *S.* Typhimurium (14). RpoS inactivation impaired growth of *S*. Typhimurium at pH 4.5. However, despite the non-functional *rpoS* in the S. Typhi Ty2 strain, no significant difference in growth at pH 4.5 was observed when *S.* Typhi CT18 carrying a functional *rpoS* allele was compared with *rpoS-*deficient *S.* Typhi Ty2, suggesting the importance of RpoS-independent factors and/or because RpoS function is not fully restored in *S.* Typhi CT18 (Fig. 4C).

Transcriptional analysis revealed the upregulation of intrinsically disordered protein (IDP)-encoding genes *yciG*, *STM14_1829*, and *ymdF* in *S.* Typhimurium with no induction detected in *S.* Typhi. The three genes exhibit a high degree of sequence similarity (80% identity), and their encoded proteins are rich in conserved, repeated hydrophilic motifs. The IDPs have been previously implicated in stress responses and flagella-dependent motility of *S.* Typhimurium (22, 24). RpoS is known to regulate the expression of these IDPs during acid stress (31). Consistent with this role, our quantitative real-time PCR analysis demonstrated a lack of induction of these IDPs in an *S.* Typhimurium Δ*rpoS* mutant (Fig. 4G). In *S.* Typhimurium, the deletion of *yciG*, *STM14_1829*, or *ymdF* did not increase the growth impairment of a *ΔrpoS* mutant strain. However, the deletion of *rpoS* and all three IDP genes (Δ*rpoS*Δ*yciG*Δ*STM14_1829*Δ*ymdF*) resulted in a greater growth impairment at pH 4.5, suggesting a contribution of additional RpoS-dependent genes to acid resistance and redundancy of function among the IDPs (Fig. 4E). Furthermore, deficient expression of the IDP homologs *STY1323*, *STY1548*, and *STY1156* in *S*. Typhi CT18 at pH 4.5 indicates that the RpoS function remains deficient in this strain despite a wild-type *rpoS* gene (Fig. 4F).

Collectively, these observations show that the reduced acid stress resistance of *S.* Typhi in comparison to *S.* Typhimurium is attributable to the contribution of multiple genetic differences, including genes encoding outer membrane proteins, a periplasmic hydrogenase, the transcriptional regulator RpoS, and stress response IDPs, which are absent or dysregulated in *S.* Typhi. Most importantly, the loss of acid resistance in *S.* Typhi by genomic decay raises the question of why this human-specific serovar no longer requires the ability to grow at pH 4.5. Part of the explanation may be the higher pH of the human large intestine in comparison to mice, the natural host of *S.* Typhimurium (42, 43). In addition, the loss of acid resistance suggests that acidification of the *S.* Typhi-containing vacuole in human phagocytes may be impaired compared to that of *S.* Typhimurium, a hypothesis that is currently being investigated.

## MATERIALS AND METHODS

### Bacterial strains and growth conditions

Bacterial strains used in this study are listed in Table S4. Bacteria were routinely grown in LB-Miller broth containing 10 g/L tryptone, 5 g/L yeast extract, and 10 g/L NaCl. LB agar contained 15 g/L Bacto agar. For experiments, bacteria were grown in 25 mL media in 250 mL Erlenmeyer flasks, unless otherwise indicated. When required, the antibiotic kanamycin (Km) was added to a final concentration of 50 μg/mL, ampicillin (Amp) to 100 μg/mL, and tetracycline (Tet) to 25 μg/mL. For pH experiments, pH of LB was adjusted using HCl or NaOH and buffered with sodium citrate (pH 3.0–4.5), MES (pH 5.0–6.0), or MOPS (pH 6.5–8.0) at a 100 mM final concentration. Unbuffered LB-Miller broth (LB-reg) had a pH of 6.8–6.9.

### RNA isolation, cDNA library preparation, and Illumina sequencing

Total RNA from *S.* Typhi Ty2 or *S*. Typhimurium 14028s grown at acidic pH 4.5 or neutral pH 7.5 was isolated using TRIzol and Zymo kits. Briefly, 1 mL of overnight bacterial culture grown in LB-reg was washed twice in either LB-citrate pH 4.5 or LB-MOPS pH 7.5, then diluted 1:100 into 25 mL of LB at the respective pH in 250 mL sterilized Erlenmeyer flasks. Cells were grown at 37°C with shaking at 200 rpm until the OD_600_ reached 0.3. Six milliliters of the cultures was mixed with 2/5th volume of ice-cold RNA-stabilizing solution (containing 5% acidic phenol and 95% ethanol) and incubated on ice for 30 min to stabilize the RNA (44). Bacteria were then pelleted and immediately solubilized with 1 mL TRIzol, which was immediately used for RNA isolation or stored at −20°C until RNA isolation. The TRIzol homogenate was processed using the Zymo extraction kit, and extracted RNA was checked for quantity using the Qubit RNA BR assay and quality using the Agilent 2100 Tapestation and RNA assay. A total of 12 RNA samples (three biological replicates of each strain grown in pH 4.5 and 7.5) were prepared for this study.

The RNA samples were sent to SeqCenter (Pittsburgh, USA) for library preparation and Illumina sequencing. Samples were DNAse treated with Invitrogen DNAse (RNAse-free). Library preparation was performed using Illumina’s Stranded Total RNA Prep Ligation with Ribo-Zero Plus Kit and 10 bp unique dual indices (UDI). Sequencing was done on a NovaSeq X Plus, producing paired-end 150 bp reads. Demultiplexing, quality control, and adapter trimming were performed with bcl-convert (v4.1.5).

### RNA-seq data analysis

The RNA-seq reads were uploaded to the Galaxy web platform and analyzed using the public server at usegalaxy.org (45). The quality of each RNA-seq library was assessed with FastQC v0.12.1 (http://www.bioinformatics.babraham.ac.uk/projects/fastqc/), and Cutadapt v4.4 (https://journal.embnet.org/index.php/embnetjournal/article/view/200) was used for processing. All reads shorter than 20 nucleotides in length after trimming were discarded from further analysis. The remaining reads of each library were aligned to the corresponding genomes (*S.* Typhi 2: NC_004631.1; *S.* Typhimurium 14028s: NC_016856.1 [chromosome] and NC_016855.1 [plasmid]) using STAR v2.7.10b (46), and alignments were filtered with Samtools v1.16.1 (47) using a MAPQ cutoff of 60. The RNA-seq mapping statistics are detailed in Table S5. Reads were assigned to genomic features using featureCounts v2.0.3 (48, 49). DESeq2 (49) was used to identify differentially expressed genes between the two pH conditions in each strain. Biological significance cutoff was set at log_2_FC ≥ 1 or log_2_FC ≤ −1 and *P*-value ≤ 0.05.

### Measurement of bacterial growth in broth

Bacterial cultures were grown overnight in LB-reg with shaking (200 rpm) at 37°C, then adjusted to OD_600_ ~1 and diluted 1:100 into LB at respective pH values with pH buffers. Each bacterial suspension was then used to inoculate the wells of a Bioscreen C honeycomb plate (Growth Curves, Ltd., Finland), each containing 300 µL of medium, at a 1:10 dilution (i.e., starting OD_600_ of 0.001). The plate was incubated at 37°C with oscillation at 600 rpm for 12 h, with OD_600_ readings taken at 15-min intervals. Wells containing non-inoculated media were included as negative controls. At least three technical replicates and three biological repeats per strain and pH level were performed.

### Real-time quantitative reverse transcription PCR (qRT-PCR)

Bacterial cultures were grown in buffered LB at pH 4.5 or 7.5 to log phase, and RNA was isolated from 2 mL of culture by TRIzol extraction (Life Technologies). RNA was quantified on a Nanovue spectrophotometer (Cytiva) and cDNA generated using the Qiagen QuantiTect Reverse Transcription Kit (Qiagen, Hilde, Germany). cDNA was quantified on a Bio-Rad CFX96 (Bio-Rad, Hercules, CA) using SYBR Green Master Mix with gene-specific primers. All qPCR assays were run in technical triplicates. Mean comparative threshold cycle (Ct) value for each transcript was adjusted for input variation by subtracting the mean *gmk* housekeeping control Ct from the target gene’s Ct to generate ΔCt (50). The ΔΔCt for each sample was calculated by subtracting target gene ΔCt in pH 7.5 cultures from target gene ΔCt in pH 4.5 cultures. Fold change in gene expression was calculated as 2^−ΔΔCt^. Primer sequences of qPCR target genes are listed in Table S6.

### Construction of deletion mutants in *S*. Typhimurium 14028s

*S.* Typhimurium deletion mutants were constructed using the λ-Red recombineering method (51, 52). Briefly, oligonucleotides listed in Table S6 were used to amplify the Km (kanamycin) resistance cassette from the pKD4 plasmid. The polymerase chain reaction (PCR) product was electroporated into 14028s cells containing the pKD46 or pSIJ8 plasmid. Transformants were selected on LB agar plates containing 50 µg/mL kanamycin at 37°C (for pKD46) or LB agar plates containing 50 µg/mL kanamycin and 100 µg/mL ampicillin at 30°C (for pSIJ8). The Km resistance cassette was removed by FLP-recombinase either by pCP20 or rhamnose induction.

### Construction of pJK770-*hydB*

The *hydB* gene was heterologously expressed in *S.* Typhi Ty2 using the constitutive expression vector pJK770. First, *hydB* was amplified using *S*. Typhimurium genomic DNA with pJK770 overlapping primers, followed by ligation with pJK770 vector digested with NcoI. The ligated product was transformed into *S.* Typhi Ty2 or S. Typhi Δ*t1450::phoE*_STm_ competent cells through electroporation, and transformants were selected on LB agar plates containing 100 µg/mL ampicillin at 37°C. *S.* Typhi colonies containing the pJK770-*hydB* plasmid were confirmed by Sanger sequencing. pJK770 vector without insert was transformed to generate *S.* Typhi Ty2 pJK770 vector.

### Allelic exchange

Replacement of S. Typhi Ty2 *t1450* with S. Typhimurium 14028s *STM14_1848* and S. Typhimurium 14028s *rpoS* with S. Typhi Ty2 *rpoS* was performed using the allelic exchange method as described in reference 53 with some modifications. The *S.* Typhi Ty2 *t1450* gene was first replaced by a *tetRA* cassette using λ-Red recombineering as described previously. Ty2 Δ*t1450:tetRA* was then used as the recipient strain in the allelic exchange protocol. For *rpoS* allelic exchange, the STy2 *rpoS* gene was cloned into the JKe201 *E. coli* strain using pFOK plasmids with 700 bp upstream and downstream regions from STm14028s. STm14028s was then used as a recipient strain for allelic exchange with JKe201-pFOK-rpoS.

### Statistical analysis

The bacterial growth rate was calculated by performing linear regression analysis on the natural logarithms of OD_600_ values of the exponential growth phase. The slopes of the growth curves were derived from three independent assays, and *P*-values were determined using an unpaired two-tailed *t*-test, with *P* ≤ 0.05 considered statistically significant.

## Data Availability

RNA-seq data have been deposited at Gene Expression Omnibus under accession no. GSE317267.

## SUPPLEMENTAL MATERIAL

Supplemental tables jb.00197-26-s0001.xlsx Tables S1 and S2.

Supplemental material jb.00197-26-s0002.docx Tables S3 to S6; Figs. S1 and S2.

ASM does not own the copyrights to Supplemental Material that may be linked to, or accessed through, an article. The authors have granted ASM a non-exclusive, world-wide license to publish the Supplemental Material files. Please contact the corresponding author directly for reuse.

## References

[1] Balasubramanian R, Im J, Lee J-S, Jeon HJ, Mogeni OD, Kim JH, Rakotozandrindrainy R, Baker S, Marks F. 2019. The global burden and epidemiology of invasive non-typhoidal *Salmonella* infections. Hum Vaccin Immunother 15:1421–1426. doi:10.1080/21645515.2018.1504717 PMC6663144 30081708

[2] Bhandari J, Thada PK, DeVos E. 2020. Typhoid fever. Stat Pearls Publishing, Treasure Island (FL).

[3] Centers for Disease Control and Prevention (CDC). 2013. Incidence and trends of infection with pathogens transmitted commonly through food - foodborne diseases active surveillance network, 10 U.S. sites, 1996-2012. MMWR Morb Mortal Wkly Rep 62:283–287. PMC4604974 23594684

[4] Murray CJ. 1991. Salmonellae in the environement. Rev Sci Tech OIE 10:765–785. doi:10.20506/rst.10.3.568 1782428

[5] Stanaway JD, Parisi A, Sarkar K, Blacker BF, Reiner RC, Hay SI, Nixon MR, Dolecek C, James SL, Mokdad AH, et al.. 2019. The global burden of non-typhoidal Salmonella invasive disease: a systematic analysis for the Global Burden of Disease Study 2017. Lancet Infect Dis 19:1312–1324. doi:10.1016/S1473-3099(19)30418-9 PMC6892270 31562022

[6] Fang FC, Frawley ER, Tapscott T, Vázquez-Torres A. 2016. Bacterial stress responses during host infection. Cell Host Microbe 20:133–143. doi:10.1016/j.chom.2016.07.009 PMC4985009 27512901

[7] Shen S, Fang FC. 2012. Integrated stress responses in *Salmonella*. Int J Food Microbiol 152:75–81. doi:10.1016/j.ijfoodmicro.2011.04.017 PMC3164900 21570144

[8] Foster JW, Spector MP. 1995. How *Salmonella* survive against the odds. Annu Rev Microbiol 49:145–174. doi:10.1146/annurev.mi.49.100195.001045 8561457

[9] Richard HT, Foster JW. 2003. Acid resistance in *Escherichia coli*. Adv Appl Microbiol 52:167–186. doi:10.1016/s0065-2164(03)01007-4 12964244

[10] Alvarez-Ordóñez A, Fernández A, Bernardo A, López M. 2010. Arginine and lysine decarboxylases and the acid tolerance response of *Salmonella* Typhimurium. Int J Food Microbiol 136:278–282. doi:10.1016/j.ijfoodmicro.2009.09.024 19864032

[11] Foster JW. 2004. *Escherichia coli* acid resistance: tales of an amateur acidophile. Nat Rev Microbiol 2:898–907. doi:10.1038/nrmicro1021 15494746

[12] Bearson BL, Wilson L, Foster JW. 1998. A low pH-inducible, PhoPQ-dependent acid tolerance response protects *Salmonella* typhimurium against inorganic acid stress. J Bacteriol 180:2409–2417. doi:10.1128/JB.180.9.2409-2417.1998 PMC107183 9573193

[13] Foster JW, Hall HK. 1992. Effect of *Salmonella* typhimurium ferric uptake regulator (fur) mutations on iron- and pH-regulated protein synthesis. J Bacteriol 174:4317–4323. doi:10.1128/jb.174.13.4317-4323.1992 PMC206215 1624426

[14] Lee IS, Lin J, Hall HK, Bearson B, Foster JW. 1995. The stationary-phase sigma factor sigma S (RpoS) is required for a sustained acid tolerance response in virulent *Salmonella* typhimurium. Mol Microbiol 17:155–167. doi:10.1111/j.1365-2958.1995.mmi_17010155.x 7476202

[15] Lee IS, Slonczewski JL, Foster JW. 1994. A low-pH-inducible, stationary-phase acid tolerance response in *Salmonella typhimurium*. J Bacteriol 176:1422–1426. doi:10.1128/jb.176.5.1422-1426.1994 PMC205208 8113183

[16] Tiwari RP, Sachdeva N, Hoondal GS, Grewal JS. 2004. Adaptive acid tolerance response in *Salmonella enterica* serovar Typhimurium and *Salmonella enterica* serovar Typhi. J Basic Microbiol 44:137–146. doi:10.1002/jobm.200310333 15069673

[17] Bäumler A, Fang FC. 2013. Host specificity of bacterial pathogens. Cold Spring Harb Perspect Med 3:a010041. doi:10.1101/cshperspect.a010041 PMC3839602 24296346

[18] Shane AL, Mody RK, Crump JA, Tarr PI, Steiner TS, Kotloff K, Langley JM, Wanke C, Warren CA, Cheng AC, Cantey J, Pickering LK. 2017. 2017 Infectious Diseases Society of America clinical practice guidelines for the diagnosis and management of infectious diarrhea. Clin Infect Dis 65:e45–e80. doi:10.1093/cid/cix669 PMC5850553 29053792

[19] Sabbagh SC, Forest CG, Lepage C, Leclerc JM, Daigle F. 2010. So similar, yet so different: uncovering distinctive features in the genomes of *Salmonella enterica* serovars Typhimurium and Typhi. FEMS Microbiol Lett 305:1–13. doi:10.1111/j.1574-6968.2010.01904.x 20146749

[20] Garai P, Gnanadhas DP, Chakravortty D. 2012. *Salmonella enterica* serovars Typhimurium and Typhi as model organisms: revealing paradigm of host-pathogen interactions. Virulence 3:377–388. doi:10.4161/viru.21087 PMC3478240 22722237

[21] Deng W, Liou S-R, Plunkett G 3rd, Mayhew GF, Rose DJ, Burland V, Kodoyianni V, Schwartz DC, Blattner FR. 2003. Comparative genomics of *Salmonella enterica* serovar Typhi strains Ty2 and CT18. J Bacteriol 185:2330–2337. doi:10.1128/JB.185.7.2330-2337.2003 PMC151493 12644504

[22] Oguri T, Kwon Y, Woo JKK, Prehna G, Lee H, Ning M, Won KJ, Lee J, Mei S, Shi Y, Jeong H, Lee H. 2019. A family of small intrinsically disordered proteins involved in flagellum-dependent motility in Salmonella enterica. J Bacteriol 201:e00415-18. doi:10.1128/JB.00415-18 PMC6304668 30373755

[23] Robbe-Saule V, Norel F. 1999. The rpoS mutant allele of *Salmonella typhi* Ty2 is identical to that of the live typhoid vaccine Ty21a. FEMS Microbiol Lett 170:141–143. doi:10.1111/j.1574-6968.1999.tb13366.x 9919662

[24] Ryan D, Pati NB, Ojha UK, Padhi C, Ray S, Jaiswal S, Singh GP, Mannala GK, Schultze T, Chakraborty T, Suar M. 2015. Global transcriptome and mutagenic analyses of the acid tolerance response of *Salmonella enterica* serovar Typhimurium. Appl Environ Microbiol 81:8054–8065. doi:10.1128/AEM.02172-15 PMC4651094 26386064

[25] Viala JPM, Méresse S, Pocachard B, Guilhon A-A, Aussel L, Barras F. 2011. Sensing and adaptation to low pH mediated by inducible amino acid decarboxylases in *Salmonella*. PLoS One 6:e22397. doi:10.1371/journal.pone.0022397 PMC3143133 21799843

[26] Boyd EF, Porwollik S, Blackmer F, McClelland M. 2003. Differences in gene content among *Salmonella enterica* serovar typhi isolates. J Clin Microbiol 41:3823–3828. doi:10.1128/JCM.41.8.3823-3828.2003 PMC179825 12904395

[27] Parkin A, Bowman L, Roessler MM, Davies RA, Palmer T, Armstrong FA, Sargent F. 2012. How *Salmonella oxidises* H(2) under aerobic conditions. FEBS Lett 586:536–544. doi:10.1016/j.febslet.2011.07.044 21827758

[28] O’Neal CR, Gabriel WM, Turk AK, Libby SJ, Fang FC, Spector MP. 1994. RpoS is necessary for both the positive and negative regulation of starvation survival genes during phosphate, carbon, and nitrogen starvation in *Salmonella typhimurium*. J Bacteriol 176:4610–4616. doi:10.1128/jb.176.15.4610-4616.1994 PMC196281 8045891

[29] Hengge-Aronis R. 2002. Signal transduction and regulatory mechanisms involved in control of the sigma(S) (RpoS) subunit of RNA polymerase. Microbiol Mol Biol Rev 66:373–395, doi:10.1128/MMBR.66.3.373-395.2002 PMC120795 12208995

[30] Zhao N, Xiang X, Liu D, Zhou K, Hu K, Li Q, Li J, Liu A, Yang Y, Ao X, Liu S. 2026. Regulatory roles of rpoS and rpoE on the response profiles of *Salmonella enterica* serovar Typhimurium to acid and osmosis sequential cross-stress treatment. Food Res Int 235:119136. doi:10.1016/j.foodres.2026.119136 42083195

[31] Lago M, Monteil V, Douche T, Guglielmini J, Criscuolo A, Maufrais C, Matondo M, Norel F. 2017. Proteome remodelling by the stress sigma factor RpoS/σ^S^ in *Salmonella*: identification of small proteins and evidence for post-transcriptional regulation. Sci Rep 7:2127. doi:10.1038/s41598-017-02362-3 PMC5437024 28522802

[32] Rathman M, Sjaastad MD, Falkow S. 1996. Acidification of phagosomes containing *Salmonella* typhimurium in murine macrophages. Infect Immun 64:2765–2773. doi:10.1128/iai.64.7.2765-2773.1996 PMC174137 8698506

[33] McConnell EL, Basit AW, Murdan S, Ibarra JA, Steele-Mortimer O. 2008. Measurements of rat and mouse gastrointestinal pH, fluid and lymphoid tissue, and implications for in-vivo experiments. J Pharm Pharmacol 60:63–70. doi:10.1211/jpp.60.1.0008 18088506

[34] Ibarra JA, Steele-Mortimer O. 2009. *Salmonella*–the ultimate insider. *Salmonella* virulence factors that modulate intracellular survival. Cell Microbiol 11:1579–1586. doi:10.1111/j.1462-5822.2009.01368.x PMC2774479 19775254

[35] Mendoza-Mejía BD, Medina-Aparicio L, Serrano-Fujarte I, Vázquez A, Calva E, Hernández-Lucas I. 2021. *Salmonella enterica* serovar Typhi genomic regions involved in low pH resistance and in invasion and replication in human macrophages. Ann Microbiol 71. doi:10.1186/s13213-021-01629-5

[36] Walsh NP, Alba BM, Bose B, Gross CA, Sauer RT. 2003. OMP peptide signals initiate the envelope-stress response by activating DegS protease via relief of inhibition mediated by its PDZ domain. Cell 113:61–71. doi:10.1016/s0092-8674(03)00203-4 12679035

[37] Harikrishnan H, Ismail A, Banga Singh KK. 2013. Temperature-regulated expression of outer membrane proteins in *Shigella flexneri*. Gut Pathog 5:38. doi:10.1186/1757-4749-5-38 PMC4029548 24330657

[38] Bekhit A, Fukamachi T, Saito H, Kobayashi H. 2011. The role of OmpC and OmpF in acidic resistance in *Escherichia coli*. Biol Pharm Bull 34:330–334. doi:10.1248/bpb.34.330 21372380

[39] Zbell AL, Benoit SL, Maier RJ. 2007. Differential expression of NiFe uptake-type hydrogenase genes in *Salmonella enterica* serovar Typhimurium. Microbiology (Reading) 153:3508–3516. doi:10.1099/mic.0.2007/009027-0 17906148

[40] Bang IS, Kim BH, Foster JW, Park YK. 2000. OmpR regulates the stationary-phase acid tolerance response of *Salmonella enterica* serovar typhimurium. J Bacteriol 182:2245–2252. doi:10.1128/JB.182.8.2245-2252.2000 PMC111274 10735868

[41] Robbe-Saule V, Algorta G, Rouilhac I, Norel F. 2003. Characterization of the RpoS status of clinical isolates of *Salmonella enterica*. Appl Environ Microbiol 69:4352–4358. doi:10.1128/AEM.69.8.4352-4358.2003 PMC169149 12902215

[42] Yamamura R, Inoue KY, Nishino K, Yamasaki S. 2023. Intestinal and fecal pH in human health. Front Microbiomes 2:1192316. doi:10.3389/frmbi.2023.1192316 PMC12993532 41853365

[43] Fallingborg J. 1999. Intraluminal pH of the human gastrointestinal tract. Dan Med Bull 46:183–196. 10421978

[44] Tedin K, Bläsi U. 1996. The RNA chain elongation rate of the lambda late mRNA is unaffected by high levels of ppGpp in the absence of amino acid starvation. J Biol Chem 271:17675–17686. doi:10.1074/jbc.271.30.17675 8663373

[45] Abueg LAL, Afgan E, Allart O, Awan AH, Bacon WA, Baker D, Bassetti M, Batut B, Bernt M, Blankenberg D, et al.. 2024. The Galaxy platform for accessible, reproducible, and collaborative data analyses: 2024 update. Nucleic Acids Res 52:W83–W94. doi:10.1093/nar/gkae410 PMC11223835 38769056

[46] Dobin A, Davis CA, Schlesinger F, Drenkow J, Zaleski C, Jha S, Batut P, Chaisson M, Gingeras TR. 2013. STAR: ultrafast universal RNA-seq aligner. Bioinformatics 29:15–21. doi:10.1093/bioinformatics/bts635 PMC3530905 23104886

[47] Danecek P, Bonfield JK, Liddle J, Marshall J, Ohan V, Pollard MO, Whitwham A, Keane T, McCarthy SA, Davies RM, Li H. 2021. Twelve years of SAMtools and BCFtools. Gigascience 10:giab008. doi:10.1093/gigascience/giab008 PMC7931819 33590861

[48] Liao Y, Smyth GK, Shi W. 2014. featureCounts: an efficient general purpose program for assigning sequence reads to genomic features. Bioinformatics 30:923–930. doi:10.1093/bioinformatics/btt656 24227677

[49] Love MI, Huber W, Anders S. 2014. Moderated estimation of fold change and dispersion for RNA-seq data with DESeq2. Genome Biol 15:550. doi:10.1186/s13059-014-0550-8 PMC4302049 25516281

[50] Ryan D, Ojha UK, Jaiswal S, Padhi C, Suar M. 2016. The small RNA DsrA influences the acid tolerance response and virulence of *Salmonella enterica* Serovar Typhimurium. Front Microbiol 7:599. doi:10.3389/fmicb.2016.00599 PMC4844625 27199929

[51] Datsenko KA, Wanner BL. 2000. One-step inactivation of chromosomal genes in *Escherichia coli* K-12 using PCR products. Proc Natl Acad Sci USA 97:6640–6645. doi:10.1073/pnas.120163297 PMC18686 10829079

[52] Jensen SI, Lennen RM, Herrgård MJ, Nielsen AT. 2015. Seven gene deletions in seven days: fast generation of *Escherichia coli* strains tolerant to acetate and osmotic stress. Sci Rep 5:17874. doi:10.1038/srep17874 PMC4672327 26643270

[53] Cianfanelli FR, Cunrath O, Bumann D. 2020. Efficient dual-negative selection for bacterial genome editing. BMC Microbiol 20:129. doi:10.1186/s12866-020-01819-2 PMC7245781 32448155

