# Supplementary material for: Impaired acid stress resistance in Salmonella Typhi Ty2
Source: J Bacteriol. 2026 Sep 23;208(10):e00197-26. doi: 10.1128/jb.00197-26 (PMC13689561; doi:10.1128/jb.00197-26)
Supplement: Supplementary file 1 [file jb.00197-26-s0002.docx]

**Impaired acid stress resistance in *Salmonella* Typhi Ty2**

Kiranmai Joshi^a^*, Wai Yee Fong^a^*, Marie-Pierre Blanc^a^,

Fermin E. Guerra^a^, and Ferric C. Fang^a,b#^

^a^Department of Laboratory Medicine and Pathology, University of Washington, Seattle, Washington, United States of America

^b^Department of Microbiology, University of Washington, Seattle, Washington, United States of America

**Supplementary Figures**

Fig S1 (A) Growth of STm wt, STm ΔompC, STm ΔompD, STm ΔompF and STm ΔphoE in buffered LB at pH 4.5. (B) Growth of STm wt, STm ΔompC ΔompD, STm ΔompD ΔphoE and STm ΔompC ΔompD ΔphoE in buffered LB at pH 4.5. (C) Growth of STm wt, STm ΔompC ΔompF, STm ΔompF ΔphoE and STm ΔompC ΔompF ΔphoE in buffered LB at pH 4.5 (D) Growth of STm wt, STm ΔompC ΔphoE and STm ΔompC ΔphoE ΔhydB in buffered LB at pH 7.5. (E) Growth of STm wt and STy::phoE_STm_ in buffered LB at pH 7.5. (F) Growth of STm wt and STm ΔhydB in buffered LB at pH 4.5. (G) Growth of STm wt, STy-Vector (pJK770) and STy-phydB (pJK770-hydB) in buffered LB at pH 7.5(H) Growth of STy wt, STy::phoE_STm,_ STy-Vector (pJK770) STy-phydB (pJK770-hydB), STy::phoE_STm_+ Vector (pJK770) and STy::phoE_STm_+ phydB (pJK770-hydB) in buffered LB at pH 7.5. No significant growth differences were observed between wildtype and mutant strains of STm and STy at pH 7.5. To generate the growth curves, bacteria were grown in LB-citrate (pH-4.5) and LB-MOPS (pH-7.5) and OD_600_ measured every 15 min at 37^o^C for 12 h on a Bioscreen C. Statistical analysis was performed in GraphPad prism 10.0. Bar graphs represent the mean slopes of exponential growth with error bars representing the standard deviation from three independent assays. P values were determined using an un-paired two-tailed t-test (B & C); ns, not significant.

**Figure S2**

Fig S2 (A) Growth of STm wt and STm ΔrpoS in buffered LB at pH 7.5. (B) Growth of STy Ty2 and STy CT18 in buffered LB at pH 7.5 (C) Growth of STm wt and STm::rpoS_STy_ in buffered LB at pH 7.5 (D) Growth of STm wt, STm ΔyciG, STm ΔSTM14_1829 and STm ΔymdF in buffered LB at pH 4.5. (E) Growth of STm wt, STm ΔyciG ΔSTM14_1829, STm ΔyciG ΔymdF, STm ΔSTM14_1829 ΔymdF and STm ΔyciG ΔSTM14_1829 ΔymdF in buffered LB at pH 4.5 (F) Growth of STm wt and STm ΔrpoS ΔyciG ΔSTM14_1829 ΔymdF in buffered LB at pH 7.5. No significant growth differences were observed between wildtype and mutant strains of STm and STy at pH 7.5 except for the quadruple mutant STm ΔrpoS ΔyciG ΔSTM14_1829 ΔymdF, which showed moderately impaired growth at neutral pH. To generate the growth curves, bacteria were grown in LB-citrate (pH-4.5) or LB-MOPS (pH-7.5) and OD_600_ measured every 15 min at 37^o^C for 12 h on a Bioscreen C. Typical growth curves representative of three independent experiments are shown. Statistical analysis was performed in GraphPad prism 10.0. Bar graphs represent the mean slopes of exponential growth with error bars representing the standard deviation from three independent assays. P values were determined using an un-paired two-tailed t-test (G); ns, not significant.

**Table S3: Transcription profiles of genes of interest**

| **Gene name** | **14028s GenBank ID** | **14028s log2(FC)** | **Ty2 GenBank ID** | **Ty2 log2(FC)** |
| --- | --- | --- | --- | --- |
| ***ompC* (*ompN*)** | STM14_1777 | 0.177 | t1341 | 1.187 |
| ***ompC* (*phoE*)** | STM14_1848 | 7.002 | t1450* | 6.463 |
| ***STM14_2797* (*ompC*)** | STM14_2797 | 0.293 | t0597 | -1.546 |
| ***ompF*** | STM14_1130 | -3.640 | t1935 | -4.638 |
| ***ompD*** | STM14_1898 | -8.760 | -- | #N/A |
| ***phoQ*** | STM14_14088 | 1.459 | t1690 | 1.149 |
| ***phoP*** | STM14_1409 | 1.179 | t1689 | 1.255 |
| ***fur*** | STM14_0808 | -0.973 | t2182 | -1.151 |
| ***ompR*** | STM14_4217 | 1.255 | t4004 | 1.549 |
| ***envZ*** | STM14_4216 | 1.364 | t4005 | 1.244 |
| ***rpoS*** | STM14_3526 | 0.822 | -- | #N/A |
| ***yciG*** | STM14_2091 | 5.720 | t1640 | -0.724 |
| ***STM14_1829*** | STM14_1829 | 4.266 | t1434 | -2.885 |
| ***ymgB*** | STM14_1529 | 2.663 | t1145 | -2.079 |
| ***ygdI*** | STM14_3597 | 2.418 | t2891 | -1.103 |
| ***iraM*** | STM14_1261 | 2.041 | t05500 | -2.531 |
| ***ymdF*** | STM14_1275 | 4.878 | t1800 | 0.602 |
| ***narW*** | STM14_1907 | 5.552 | t1490 | 2.410 |
| ***narV*** | STM14_1908 | 2.636 | -- | #N/A |
| ***hydB*** | STM14_1856 | 4.014 | -- | #N/A |
| ***fdhN*** | STM14_1894 | -0.177 | -- | #N/A |
| ***adiA*** | STM14_5169 | 3.00 | t4203 | 2.81 |
| ***adiC*** | STM14_5166 | 2.62 | t4201 | 1.30 |
| ***speF*** | STM14_0818 | 1.18 | t2174 | 0.69 |
| ***speC*** | STM14_3761 | 1.212 | -- | #N/A |
| ***cadA*** | STM14_3138 | 5.09 | t0297 | 4.19 |

**Table S4: Strains, Plasmids & Reagents**

| **Bacterial Strains** | | |
| --- | --- | --- |
| **Strain** | **Reference** | **Identifier** |
| Salmonella enterica serovar Typhimurium 14028s wild type | S. Miller | JK1324, LAS5, TAS8 |
| S. Typhimurium 14028s ∆*ompC*::FRTaphFRT | This study | WYF47 |
| S. Typhimurium 14028s ∆*ompD*::FRTaphFRT | This study | WYF46 |
| S. Typhimurium 14028s ∆*ompC* ∆*ompD*::FRTaphFRT | This study | WYF76 |
| S. Typhimurium 14028s ∆*ompF*::FRTaphFRT | This study | WYF22 |
| S. Typhimurium 14028s ∆*phoE*::FRTaphFRT | This study | WYF20 |
| S. Typhimurium 14028s ∆*ompC* ∆*ompF*::FRTaphFRT | This study | WYF51 |
| S. Typhimurium 14028s ∆*ompC* ∆*phoE*::FRTaphFRT | This study | WYF50 |
| S. Typhimurium 14028s ∆*phoE* ∆*ompF*::FRTaphFRT | This study | WYF29 |
| S. Typhimurium 14028s ∆*phoE* ∆*ompD*::FRTaphFRT | This study | WYF74 |
| S. Typhimurium 14028s ∆*ompC* ∆*ompD* ∆*phoE*::FRTaphFRT | This study | WYF78 |
| S. Typhimurium 14028s ∆*ompC* ∆*ompF* ∆*phoE*::FRTaphFRT | This study | WYF57 |
| S. Typhimurium 14028s ∆*phoE* ∆*ompD*::FRTaphFRT | This study | WYF74 |
| S. Typhimurium 14028s ∆*ompC* ∆*phoE* ∆*hydB*::FRTaphFRT | This study | KRN41 |
| S. Typhimurium 14028s ∆*yciG*::FRTaphFRT | This study | WYF48 |
| S. Typhimurium 14028s ∆*ST14_1829*::FRTaphFRT | This study | WYF55 |
| S. Typhimurium 14028s ∆*ymdF*::FRTaphFRT | This study | KRN28 |
| S. Typhimurium 14028s ∆*yciG* ∆*STM14_1829*::FRTaphFRT | This study | KRN18 |
| S. Typhimurium 14028s ∆*yciG* ∆*ymdF*::FRTaphFRT | This study | KRN29 |
| S. Typhimurium 14028s ∆*STM14_1829* ∆*ymdF*::FRTaphFRT | This study | KRN30 |
| S. Typhimurium 14028s ∆*yciG* ∆*STM14_1829* ∆*ymdF*::FRTaphFRT | This study | KRN31 |
| S. Typhimurium 14028s ∆*rpoS*::tetRA | JK1269 | (1) |
| S. Typhimurium 14028s ∆*rpoS* ∆*yciG*::FRTaphFRT | This study | KRN73 |
| S. Typhimurium 14028s ∆*rpoS* ∆*yciG* ∆*STM14_1829*::FRTaphFRT | This study | KRN70 |
| S. Typhimurium 14028s ∆*rpoS* ∆*yciG* ∆*ymdF*::FRTaphFRT | This study | KRN57 |
| S. Typhimurium 14028s ∆*rpoS* ∆*ymdF* ∆*STM14_1829*::FRTaphFRT | This study | KRN71 |
| S. Typhimurium 14028s ∆*rpoS* ∆*yciG* ∆*STM14_1829* ∆*ymdF*::FRTaphFRT | This study | KRN72 |
| S. S. Typhimurium 14028s: STm::*rpoS*_STy_ | This study | KRN74 |
| STy-pJK770 | This study | TY772 |
| STy-pJK770-*hydB* | This study | TY777 |
| STy ∆*t1450*::*phoE*_STm_ | This study | TY743 |
| STy ∆*t1450*::*phoE*_STm_ + pJK770 | This study | TY891 |
| STy ∆*t1450*::*phoE*_STm_ + pJK770-*hydB* | This study | TY892 |
| Salmonella enterica serovar Typhi Ty2 wild-type JSG624 | J. Gunn | TY370 |
| **Plasmids** | | |
| Recombineering plasmid encoding kanamycin resistance cassette | pKD4 | (2) |
| λ-Red recombinase expression plasmid | pKD46 | (2) |
| pRB3-273C-pTRC-STMspvD bla | pJK770 | (3) |
| pFRC00B-derived suicide vector containing the *sacB* gene driven by *P*_tetA_ for AHT-inducible sucrose counter-selection in addition to AHT-inducible I-sceI expression | pFOK | (4) |
| **Reagents** | | |
| LB Broth, Miller, | Fisher BioReagents | Cat#BP1426 |
| LB Agar, Miller | Fisher BioReagents | Cat#BP1425 |
| Phosphate-buffered saline | Corning | Cat#21-040-CV |
| Ampicillin, sodium salt | Research Products International | Cat#A40040 |
| Kanamycin sulfate | VWR | Cat#0408 |
| Chloramphenicol | Sigma | Cat#C0378 |
| Sodium Citrate | Fisher Scientific | BP327-1 |
| MOPS | Sigma | Cat#3183 |
| MES | Sigma | Cat#M3885 |
| Trizol Reagent | Life Technologies | Cat#15596026 |
| QuantiTect Reverse Transcription kit | Qiagen | Cat#205311 |
| SYBR Green Master Mix | Bio-rad | Cat#1725274 |

**Table S5: RNA-Seq mapping statistics**

| **Sample Name** | **Total number of**  **reads sequenced** | **Total number of reads post trimming** | **Total number of reads mapped to reference** | **Number of reads uniquely mapping to reference** | **Uniquely mapped reads (%)** |
| --- | --- | --- | --- | --- | --- |
| 14028s_pH45_1 | 9,328,632 | 9,265,257 | 7,002,047 | 6,687,631 | 72.18% |
| 14028s_pH45_2 | 12,484,742 | 12,415,847 | 9,725,948 | 9,293,992 | 74.86% |
| 14028s_pH45_3 | 11,583,979 | 11,520,997 | 8,961,380 | 8,500,740 | 73.78% |
| 14028s_pH75_1 | 10,180,805 | 10,059,255 | 7,394,592 | 6,875,833 | 68.35% |
| 14028s_pH75_2 | 8,573,512 | 8,353,051 | 5,240,373 | 4,136,985 | 49.53% |
| 14028s_pH75_3 | 8,295,312 | 8,197,004 | 6,117,138 | 5,663,511 | 69.09% |
| Ty2_pH45_1 | 9,694,754 | 9,506,540 | 6,313,759 | 5,563,097 | 58.52% |
| Ty2_pH45_2 | 10,827,558 | 10,732,726 | 8,060,707 | 7,726,234 | 71.99% |
| Ty2_pH45_3 | 12,858,590 | 12,780,806 | 9,536,740 | 9,254,996 | 72.41% |
| Ty2_pH75_1 | 12,291,675 | 12,161,666 | 9,033,484 | 8,254,552 | 67.87% |
| Ty2_pH75_2 | 11,647,293 | 11,521,340 | 8,159,869 | 7,637,167 | 66.29% |
| Ty2_pH75_3 | 10,050,160 | 9,866,955 | 6,691,175 | 5,802,498 | 58.81% |

**Table S6: Oligos used in the study**

| **Primer** | **Sequence 5’-3’** | **Purpose** |
| --- | --- | --- |
| *ompC*_ko_F | AAGCAGTGGCATAAAAAAGCAATAAAGGCATATAACAGAGGGTTAATAACGTGTAGGCTGGAGCTGCTTC | Lambda red primers for deletion of ompC in STm |
| *ompC*_ko_R | TAAGGCATGAAAAAAGGGCCCGCAGGCCCTTTAGCAACATCTTTTGCTGACATATGAATATCCTCCTTAG | Lambda red primers for deletion of ompC in STm |
| *ompD*_ko_F | TTTAACAATGGTTGAGGAAACACGCTAAGAAAATTATAAGGATTATTAAAGTGTAGGCTGGAGCTGCTTC | Lambda red primers for deletion of ompD in STm |
| *ompD*_ko_R | GAAAAAGCCAGCCCTGAAAGGACTGGCTTTGTATTCAGACTACAACAAAACATATGAATATCCTCCTTAG | Lambda red primers for deletion of ompD in STm |
| *ompF*_ko_F | TGACGGCAGTGGCAGGTGTCATATAAAAAAACCAATGAGGGTAATAAATAGTGTAGGCTGGAGCTGCTTC | Lambda red primers for deletion of ompF in STm |
| *ompF*_ko_R | AACAGGACCGAAGTCCTGTTTTTGAGGCATAAAACAAAGGGGTCTGCTGACATATGAATATCCTCCTTAG | Lambda red primers for deletion of ompF in STm |
| *phoE*_ko_F | TAAGGCATATAAAATGAAAAAAGTAGTGGTGTTATCTG | Lambda red primers for deletion of phoE in STm |
| *phoE*_ko_R | AGACTGACGACGTCAAAACTCGTAGACTAAGCC | Lambda red primers for deletion of phoE in STm |
| *yciG*_ko_F | ACACGGTTAAAACCGATTTTTTCAGCAAGCAACGAGACAGGAGAAATAATGTGTAGGCTGGAGCTGCTTC | Lambda red primers for deletion of yciG in STm |
| *yciG*_ko_R | ACTCGCAGATTGCTTGATAAAAGCATGTGTTATATTTACATTACAGTAAACATATGAATATCCTCCTTAG | Lambda red primers for deletion of yciG in STm |
| *STm14_1829*_  ko_F | TGAGAAGACATATCATATTTAAAACGCAACATCATCATGAGGATTATATTGTGTAGGCTGGAGCTGCTTC | Lambda red primers for deletion of STm14_1829 in STm |
| *STm14_1829*_  ko_R | ATCGTATGTGCAATATTTCAGAATATATTTATGAAATATCAGCAGTAAAACATATGAATATCCTCCTTAG | Lambda red primers for deletion of STm14_1829 in STm |
| *ymdF*_ko_F | CCTATGCAATATGATGTCTAATCTATGACGGAGGTCAGTAGTGTAGGCTGGAGCTGCTTC | Lambda red primers for deletion of ymdF in STm |
| *ymdF*_ko_R | TTAGCGCGAAACGCTCAGCGTCTTGCTGTTGTGACGCAGGCATATGAATATCCTCCTTAG | Lambda red primers for deletion of ymdF in STm |
| *rpoS*_700up_F | TATCGATAAGCTTGATATCGCATCCGGTACGCCAATTACTG | For amplification of 700 bp upstream region of *rpoS* (STm) for Gibson assembly |
| *rpoS*-700up_R | TCTGACTCATAAGGTGGCTCCTACCCGTG | For amplification of 700 bp upstream region of *rpoS* (STm) for Gibson assembly |
| *rpoS*-700down_F | TACGGCGTAACGGTTCGGTATCGCGCC | For amplification of 700 bp downstream region of *rpoS* (STm) for Gibson assembly |
| *rpoS*-700down_R | CGGCCGCTCTAGAACTAGTGGTGGTGCGTATCGATAAGGTCTC | For amplification of 700 bp downstream region of *rpoS* (STm) for Gibson assembly |
| *rpoS*_F | GAGCCACCTTATGAGTCAGAATACGCTGAAAGTTCATG | For amplification of *rpoS* (STy) for Gibson assembly |
| *rpoS*_R | TACCGAACCGTTACGCCGTACCAGCCGC | For amplification of *rpoS* (STy) for Gibson assembly |
| *t1450*_tetR | CGAGCAGGTTTTGCGGAAAGTCCAAAAATAAGACAAATAAGGCATATAAATTAAGACCCACTTTCACATT | Lambda red primers for *t1450* replacement with tetRA |
| *t1450*_tetA | TAGTGGAAACCGCGCGAGGATGGTTAGTTCATCAGGCCAGACTGACGACGCTAAGCACTTGTCTCCTG | Lambda red primers for *t1450* replacement with tetRA |
| *t1450*_900up_F | GACGGTATCGATAAGCTTGATATCGTGCCTTATCTATTGAATAAAGGTAAC | For amplification of 900 bp upstream region of *t1450* for Gibson assembly |
| *t1450*_900up_R | TACTTTTTTCATTTTATATGCCTTATTTGTCTTATTTTTGG | For amplification of 900 bp upstream region of *t1450* for Gibson assembly |
| *t1450*_900down_F | CTACGAGTTTTGACGTCGTCAGTCTGGCCTG | For amplification of 900 bp downstream region of *t1450* for Gibson assembly |
| *t1450*_900down_R | GGTGGCGGCCGCTCTAGAACTAGTGGGTGCGCATGGAGTGCAAAG | For amplification of 900 bp downstream region of *t1450* for Gibson assembly |
| *phoE*_F | TAAGGCATATAAAATGAAAAAAGTAGTGGTGTTATCTG | For amplification of *phoE* for Gibson assembly |
| *phoE*_R | AGACTGACGACGTCAAAACTCGTAGACTAAGCC | For amplification of *phoE* for Gibson assembly |
| *rpoD* qRT-PCR_F | TTCCAGCAGATAGGTAATGGCTTC | For quantifying *rpoD* expression |
| *rpoD* qRT-PCR_R | GTGAATGGGCACTGTTGAACTG | For quantifying *rpoD* expression |
| *gmk* qRT-PCR_F | AGCAAATTCGCGAAAAGATG | For quantifying *gmk* expression |
| *gmk* qRT-PCR_R | TGGCAATGACTTCTTCGCTAT | For quantifying *gmk* expression |
| *yciG* qRT-PCR_F | AAA AGC ATC TGA AGC GGG C | For quantifying *yciG* expression |
| *yciG* qRT-PCR_R | CCG TGA CTA TTC TGA CCA CC | For quantifying *yciG* expression |
| *ymdF* qRT-PCR_F | GGC GGT TCC GGT AAT TTT G | For quantifying *ymdF* expression |
| *ymdF* qRT-PCR_R | CCC CTT TTT TGC CTG CTT C | For quantifying *ymdF* expression |
| *STM14_1829*  qRT-PCR_F | GGC GGT TCC GGT AAT TTT G | For quantifying *STM14_1829* expression |
| *STM14_1829*  qRT-PCR_R | TTG CCC CCT TTT TTG CCT G | For quantifying STM*14_1829* expression |

**References**

1.	Singletary LA, Karlinsey JE, Libby SJ, Mooney JP, Lokken KL, Tsolis RM, Byndloss MX, Hirao LA, Gaulke CA, Crawford RW, Dandekar S, Kingsley RA, Msefula CL, Heyderman RS, Fang FC. 2016. Loss of Multicellular Behavior in Epidemic African Nontyphoidal *Salmonella enterica* Serovar Typhimurium ST313 Strain D23580. mBio 7:e02265.<https://doi.org/10.1128/mbio.02265-15>

2.	Datsenko KA, Wanner BL. 2000. One-step inactivation of chromosomal genes in *Escherichia coli* K-12 using PCR products. Proc Natl Acad Sci U S A 97:6640-5.<https://doi.org/10.1073/pnas.120163297>

3.	Stepien TA, Singletary LA, Guerra FE, Karlinsey JE, Libby SJ, Jaslow SL, Gaggioli MR, Gibbs KD, Ko DC, Brehm MA, Greiner DL, Shultz LD, Fang FC. 2024. Nuclear factor kappa B-dependent persistence of *Salmonella* Typhi and Paratyphi in human macrophages. mBio 15:e0045424.<https://doi.org/10.1128/mbio.00454-24>

4.	Cianfanelli FR, Cunrath O, Bumann D. 2020. Efficient dual-negative selection for bacterial genome editing. BMC Microbiol 20:129.<https://doi.org/10.1186/s12866-020-01819-2>
